# IQ and mental health are vital predictors of work drop out and early mortality. Multi-state analyses of Norwegian male conscripts

**DOI:** 10.1371/journal.pone.0180737

**Published:** 2017-07-06

**Authors:** Stein Atle Lie, Torill H. Tveito, Silje E. Reme, Hege R. Eriksen

**Affiliations:** 1 Uni Research Health, Bergen, Norway; 2 Department of Clinical Dentistry, University of Bergen, Bergen, Norway; 3 Department of Health, Social, and Welfare studies, University College of Southeast Norway, Horten, Norway; 4 Department of Psychology, Faculty of Social Sciences, University of Oslo, Oslo, Norway; 5 Department of Sport and Physical Activity, Western Norway University of Applied Sciences, Bergen, Norway; IRCCS E. Medea, ITALY

## Abstract

**Background:**

Disability benefits and sick leave benefits represents huge costs in western countries. The pathways and prognostic factors for receiving these benefits seen in recent years are complex and manifold. We postulate that mental health and IQ, both alone and concurrent, influence subsequent employment status, disability benefits and mortality.

**Methods:**

A cohort of 918 888 Norwegian men was followed for 16 years from the age of 20 to 55. Risk for health benefits, emigration, and mortality were studied. Indicators of mental health and IQ at military enrolment were used as potential risk factors. Multi-state models were used to analyze transitions between employment, sick leave, time limited benefits, disability benefits, emigration, and mortality.

**Results:**

During follow up, there were a total of 3 908 397 transitions between employment and different health benefits, plus 12 607 deaths. Men with low IQ (below 85), without any mental health problems at military enrolment, had an increased probability of receiving disability benefits before the age of 35 (HRR = 4.06, 95% CI: 3.88–4.26) compared to men with average IQ (85 to 115) and no mental health problems. For men with both low IQ and mental health problems, there was an excessive probability of receiving disability benefits before the age of 35 (HRR = 14.37, 95% CI: 13.59–15.19), as well as an increased probability for time limited benefits and death before the age of 35 compared to men with average IQ (85 to 115) and no mental health problems.

**Conclusion:**

Low IQ and mental health problems are strong predictors of future disability benefits and early mortality for young men.

## Introduction

Disability and long-term sick leave benefits are major economic burdens in many western countries[1], and the increase in disability benefits has been attributed to mental health problems[2–4]. Mental health problems in early life have been found to increase the risk of long term sick leave[5] and disability benefits later in life[6–9]. Additionally, sick leave, unemployment, and rehabilitation benefits themselves are risk factors for later permanent disability benefits and mortality[10–12].

Besides the personal and societal economic consequences of being on the outside of the labour market, there are negative health consequences for the individual[13, 14]. Those excluded from the labour market have both higher mortality and morbidity[13].

Elucidating the lifetime course of employment, sick leave, medical rehabilitation, vocational rehabilitation, disability benefits, emigration, and early death is the goal in this study. Exploring trajectories of employment and benefits for men with mental health and IQ challenges, may elicit important knowledge for authorities when dealing with demographic transitions, since a high employment rate is crucial for maintaining the welfare system. IQ is a known factor for adverse outcomes[15, 16]. However, studies on IQ in conjunction with mental health problems are lacking. The aim of the study was to investigate if men’s mental health and IQ at the age of 18 to 20 years predicted subsequent employment, health benefits, and early mortality between 20 and 55 years of age.

## Material and methods

This study included 918 888 Norwegian men born between 1950 and 1980. As part of the selection of personnel to the Norwegian military service, all meet one day before a draft board and a military physician for assessments of physical and mental health.

Norway has compulsory military service for men between the ages of 19 and 44. In October 2014, Norway extended compulsory military service to women. Data on women are not included here. Before military service all young men are obliged to meet before a draft board for a military physician to assess their medical and psychological suitability, including general ability, for military service. Young men with severe mental disorders or with intellectual disabilities would not be included in the cohort as they would be too disabled to be considered for military duty. More than 95% of the men being evaluated are between the age of 18 and 20 years. For the general evaluation of being fit for military service, several parameters are considered. For the physical parameters, the grading ranges from 1 to 9 (Table 1). A value of 9 means no impairment, for performing military service. Two or more scores below 4 will in general classify the individual as unfit for military service. Particular qualifications (e.g. language or driving licenses) or weaknesses (e.g. drug abuse) may alter the evaluation of fit or not. Due to a different grading system before 1980, mental health data, and the physical parameters, before this time is missing.

**Table 1:** Number of individuals scored on health related variables at military enrolment (N = 918 888).

	1&2^a^	3	4	5	6	7	9	Missing
Variables	n	n	n	n	n	n	n	N
**Mental health**	6660	6998	2755	2804	5102	9561	576414	308594
**Physical health**	10104	10300	7630	11060	20638	33839	516700	308617
**Arm**	1009	6450	1069	1732	2888	4587	591358	309795
**Hand**	1082	6397	1136	1712	3065	4860	590846	309790
**Walking**	2335	7279	2702	5447	12383	27247	551814	309681
**Back**	2127	6940	2486	4583	13574	26440	553037	309701
**Skin**	1273	6427	2095	3130	6332	12671	577164	309796

a: The categories 1 & 2 were merged since category 2 only had few (<5) number of observations for all variables.

A value of 9 means no impairment, while value 8 was not used. A low score means that the impairment is serious with regards to perform military service.

### Mental health assessment

The examining physician assessed mental health on a scale from 1 to 9 (Table 1). A score of 9 indicates that the physician discovered no mental health problems. A score of 9, implying no impairment, was used if no factors were revealed that could negatively affect the conscript's ability to learn and perform military routines and operations. If a mental health problem was discovered, a score of less than 9 was applied, while a score of 8 was not applied. Conscripts were scored based on the severity of the mental health problem with regards to the ability to perform military service. Specific personality disorders (ICD-10, F60) would for instance result in a score of 6–7 if the evaluating doctor considered the condition to be minor, while a score of 1 was used if a mental health problem or disorder with a significant degree of disability were detected. A score of 6 would normally result in discharge from military service. The score 8 was not applicable for the mental health scoring[17]. See Fadum et al. for a more an elaborate description of the mental health assessment and procedure [18]. For the discussion of mental health in this article it is crucial to consider that this is not based on a verified mental condition, but merely mental problems determined with the purpose of performing military service.

### General ability level (IQ)

The general ability level was based on an IQ test and was scored on a scale from 1 to 9, using “STANINE” (STAndard NINE). The men were tested for arithmetic, figures, and word similarities on a written test[19]. The STANINE scores, with a mean of 5 and standard deviation of 2, can be transformed into IQ equivalents with a mean of 100 and standard deviation of 15[20]. If no IQ-test was taken, or the test was overridden, grades A (assumed above average), B (assumed average), or C (assumed below average) were set. Scores 1 and 2, and grade C were considered low IQ (low general ability level), corresponding to an IQ of 85 or lower. Scores 3 to 7, and grade B, corresponds to an IQ between 85 and 115, and were considered average IQ. High IQ, above 115, was defined as scores 8 and 9, and grade A (Table 2).

**Table 2:** Distribution of IQ Scores. If an IQ-test was taken, the Stanine score were present. Five is considered mean IQ, while 9 is the highest IQ. If no IQ test was taken the scores A, B and C were used. The two different IQ measures were merged into 1 combined score.

IQ test taken			No IQ test taken			Combined score		
Stanine Score	N	Percent	Score	N	Percent	Score	N	Percent
1	15 709	1,9%	C (assumed below average)	3 769	14,9%	Assumed below average^a^	57 092	6,6%
2	37 614	4,5%	B (assumed average)	20 318	80,6%	Assumed average^b^	691 580	80,1%
3	75 441	9,0%	A (assumed above average)	1 124	4,5%	Assumed above average^c^	114 697	13,3%
4	134 404	16,0%						
5	172 954	20,6%						
6	163 404	19,5%						
7	125 059	14,9%						
8	75 018	9,0%						
9	38 555	4,6%				Missing	80 730	
**Total**	**838 158**		**Total**	**25 211**		**Total**	**918 888**	

a 1, 2, and C

b 3, 4, 5, 6, 7, and B

c 8, 9, and A

### Early mortality, emigration, sick leave, and disability benefits

The Norwegian public insurance system covers all Norwegian citizens and provides health service benefits and pensions, administered by the Norwegian Welfare and Labour Administration (NAV). Sick leave compensation provides 100% coverage for lost income from day one up to 52 weeks. After that, long-term benefits (vocational rehabilitation, medical rehabilitation and, finally, disability benefits) provide approximately 66% of the former income. Norway has a targeted disability benefit granted to persons below 35 years of age, unable to support themselves because of serious mental or physical disability. This benefit has a higher payment for the total life span than the minimum benefit these persons would otherwise have received.

**Fig 1:**
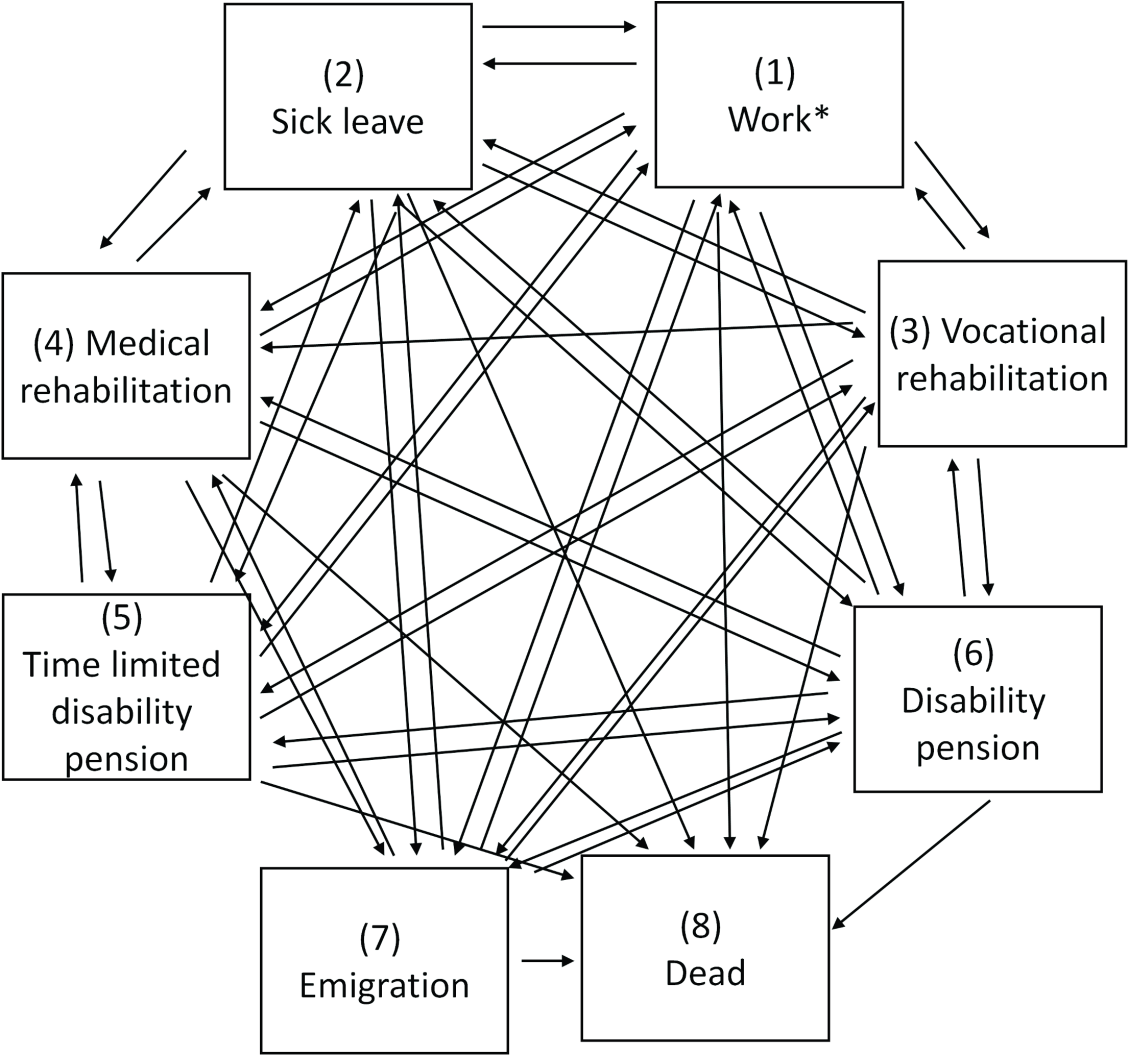
Illustration of the multi-state model with all observed states (boxes) and transitions (arrows). The state dead (8) is absorbing with no arrows out.

**Fig 2:**
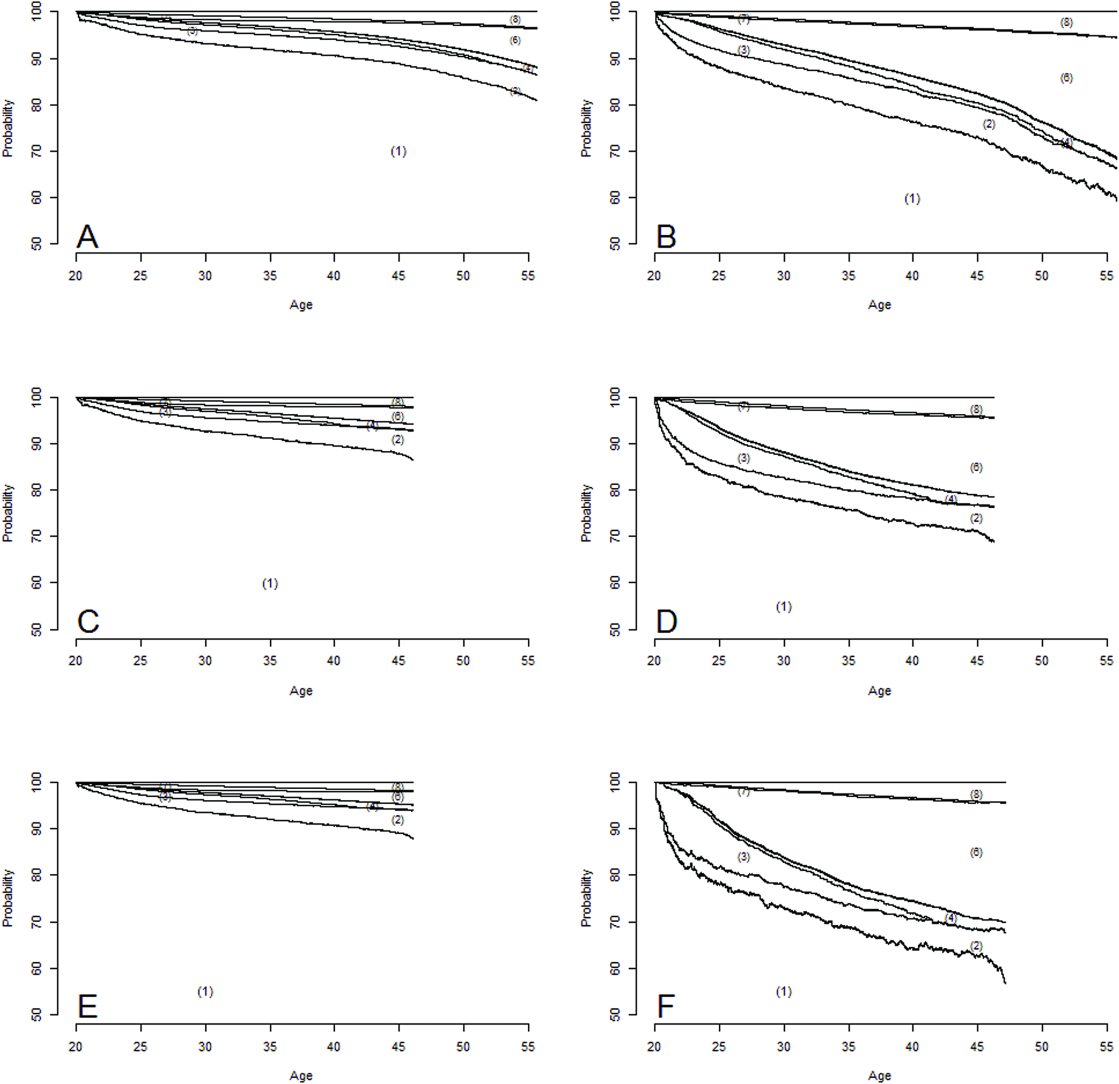
Estimated state probabilities for the states in Fig 1; (1) Employment, (2) Sick leave, (3) Vocational rehabilitation, (4) Medical rehabilitation, (5) Time limited disability benefits, (6) Disability benefits, (7) Emigrated, and (8) Dead. **A** is for 806 277 men with average to high IQ. **B** is for 57 092 men with low IQ. **C** is for 576 414 men without mental health problems. **D** is for 33 880 men with mental health problems. **E** is for 518 852 men without mental problems and average to high IQ. **F** is for the 8 130 men with mental problems and a low IQ.

The military conscription data was merged with national reimbursement data from the Norwegian Welfare and Labour Administration, obtained from the database FD-Trygd (http://www.ssb.no/a/fd-trygd/), using the unique Norwegian person identification. All sick leave episodes, reimbursement data, emigration, and mortality were available for the years between 1992 and 2008. Hence, each man contributes with different age spans, with a maximum of 16 years of follow-up. In Fig 1 and Fig 2, the states medical rehabilitation, vocational rehabilitation, and time limited disability benefits were kept separate. For the estimation of mean number of years and in the regression models these states were merged into *time limited benefits*. Multiple episodes of the transient reimbursements (sick leave, time limited benefits, and disability benefits), employment, and emigration are possible. Emigration was defined as change from a domestic address to an address abroad. Transitions between states observed with less than 10 events were removed from the analyses.

Employment was defined as time gaps where no benefit was registered, and does also include men under education. In addition, some men could also be unemployed. Since unemployment rates in Norway were low from 1992 to 2008 (from 1.7 to 5.4%, www.nav.no), we consider our definition of employment to be sufficient for the current analyses.

## Statistical methods

In order to examine the lifetime course of employment, sick leave, disability benefits, emigration, and mortality, a multi-state model was constructed [21–25] (Fig 1).

In the multi-state model the transitions (shifts) between the different states can be expressed by hazard functions for all possible transitions (arrows). The definition of the hazard functions within a multi-state framework is the hazard (intensity) for an individual to shift to state k immediately after time t (t+dt), conditioned that the individual were in state j at time t.

$${\text{h}}_{\text{jk}}(\text{t})=\underset{\text{dt}\to 0}{\lim}\frac{\text{P}\left(\text{X}(\text{t}+\text{dt})=\text{k}|\text{X}(\text{t-})=\text{j}\right)}{\text{dt}}$$ (1)

Thus, our observation, X, is that an individual is at risk in state j at time t, and then shifts to state k at time t+dt. The main objective of multi-state models is that the transitions between the different states cannot be studied independent of each other. There are for instance several paths from sick leave to work, either the direct path or via one or several of the other states, and these transitions can happen repeatedly.

To maintain that individuals may proceed in multiple paths, we define the transitions intensity matrix **h**(t). The non-diagonal elements (j≠k) in **h**(t) is the hazard corresponding to the given path from state j to state k, while the diagonal elements, h_jj_(t) in the matrix is minus the row sum of the non-diagonal elements $h_{jj}(t)=-\sum_{j\neq k}h_{jk}(t)$. Thus, the transition intensity matrix can be interpreted as the instant risk to shift (or not) from one state to another. Estimation of empirical cumulative transition intensities is often done using the non-parametric Nelson-Aalen estimator. Within multi-state models, the Nelson-Aalen estimator can be defined as

$${\hat{H}}_{jk}(t)=\sum_{T_{s}\leq t}\frac{n_{jk}(T_{s})}{Y_{j}(T_{s})}.$$ (2)

The estimate for the variance of the Nelson-Aalen estimator is

$$\hat{V}ar\left(H_{jk}(t)\right)=\sum_{T_{s}\leq t}\frac{n_{jk}(T_{s})}{{\left(Y_{j}(T_{s})\right)}^{2}}$$ (3)

In this formula n_jk_(t) is the number of shifts (events) from state j to state k at time t, while Y_j_(t) is the number of observations (individuals) in state j at time (t). Thus, Y_j_(t) defines the risk set for all who may have a transition from state j to state k at time t. T_1_<T_2_<… are the sorted times of events for an observed transition from state j to state k. The transition intensity estimates (H_jk_(t)) forms the basis in the cumulated (integrated) transition intensity matrix **H**(t), which now comprise all possible transitions in Fig 2.

Transition intensities can be of interest in itself, but gives no measure for being in the different states. We therefore want to express the probability to observe an individual in state k at time t_2_, conditioned that the individual was observed in state j at time t_1_.

$${\text{P}}_{\text{jk}}({\text{t}}_{1},{\text{t}}_{2})=\text{P}\left(\text{X}({\text{t}}_{2})=\text{k}|\text{X}({\text{t}}_{1})=\text{j}\right)$$ (4)

This probability depends on where (in which states) the individual may have been in, and the intensity/hazard to shift between these states, between time t_1_ and t_2_. The probabilities P_jk_(t_1_,t_2_) is set up in a transition probability matrix **P**(t_1_,t_2_). In the probability matrix, the sum of all row elements is 1 (100%).

The transition probabilities, often estimated non-parametric using the Aalen-Johansen estimator, are based on the transition intensities[26].

$$\hat{P}(t_{1},t_{2})=\prod_{s=t_{1}}^{t_{2}}\left[I+\Delta \hat{H}(s)\right]$$ (5)

This calculation of this product limit estimator assumes that the multi-state model is Markovian. This means that the probability to shift from one state to another at a time does not depend on the history, e.g. the time the individual has been in the current state. If the multi-state model is not Markovian additional states can be defined to define the non-Markovian patterns, or alternative methods can be used [27, 28].

The transition probabilities P(t_1_,t_2_) may often be of particular interest, e.g. what is the probability to be in work at time t_2_, conditioned that the individual was on sick-leave at time t_1_. However, the unconditional state occupation probabilities are often of more interest than the transition probabilities.

$${\text{Q}}_{\text{k}}(\text{t})=\text{P}\left(\text{X}(\text{t})=\text{k}\right)$$ (6)

The state occupation probabilities, which are the unconditional probability of being in a state, are generally estimated as a weighted sum of the columns in the transition probability matrix. The null distribution Q_k_(0) (the distribution of the k states at start of the follow-up) are typically used for weighting (W_k_).

$${\hat{Q}}_{k}(t)=\sum_{j=1}^{J}W_{j}\cdot {\hat{P}}_{jk}(0,t)$$ (7)

For calculation of present state probabilities, we defined that all men at the age of 20 years were in the state employment (which also will include education). This forces the state probabilities to start at 100% for employment at 20 years of age. If the Markovian assumption is wrong, the state occupation probabilities will still give reliable estimates, while the transition probabilities are more susceptible to the violation of the assumption. This has been shown by several authors[28, 29].

Based on the state occupation probabilities, restricted mean times (E_k_(t)) individuals spend in a given state can be calculated, integrating the state occupation probabilities.

$${\hat{E}}_{k}(t)=\int_{0}^{t}{\hat{Q}}_{k}(u)du$$ (8)

In analyses of sick leave data, numbers of sick leave days are often summed, before analyses (e.g. using linear models) are performed. Summing of sick leave days will in the presence of censoring lead to inaccurate estimation of mean number of sick leave days (and other measures). The calculation of the state expectancy, based on formula (7), account for this.

Inference (confidence intervals) for the different measures was calculated based on simulations. For the simulations 1000 replications of the transition intensity matrix using the Nelson-Aalen estimator, and its variance estimator, were applied. For the analyses of state occupation probabilities a generalized estimation equations (GEE) model was set up using a discrete time model for binary data with a complementary log-log link.

$$\log\left(-\log\left(Q_{ji}\right)\right)=\alpha_{j}+\beta^{T}\cdot Z_{i},\;j=20,\ldots ,58$$ (9)

In this model indicators for each of the states for each year of observation were constructed. A finer grid, e.g. 1 month, could be applied, but since the model is only intended to give overall average effects of the covariates, and the size of data matrix would expand substantially by doing this, one year indicator was considered sufficient. For the model we assume that each year/time has a separate intercept αα_j_. The result from this model is presented as hazard ratios. Since the measurements were balanced in time an autoregressive AR(1) correlation structure was applied for the estimation in the GEE model. More elaborate discrete time models applied to employment data has been discussed[30]. Alternatively to the discrete time approach, pseudo data could have been generated and used in the GEE model[31, 32].

Since the last age to apply for the targeted disability pension at young age in Norway was 35 years, the effect before and after 35 years of age were studied in the regression models. In the adjusted regression models, adjustment was done using the available physical variables. All the physical variables were dichotomized as 9 or below, while BM was used as a continuous variable.

The discrete time regression models were set up in the statistical package Stata (StataCorp. 2015. Release 14. TX), while the remaining calculations were performed in “R” (https://www.r-project.org/). P-values less than 0.05 were considered statistically significant.

## Results

A total of 3 921 004 transitions between the different states during the observation period were observed (Table 3). The mean number of transitions was 4.2, while the median number was 1 (quartile range: 0–6, range: 0–235). The highest state probability, except for employment, at the age of 35, was observed for sick leave; 3.1% for the 806 277 men with average to high IQ (Fig 2A), 3.4% for the 576 414 men without mental health problems (Fig 2C), and 3.3% for the 518 852 men with average to high IQ with no mental health problem (Fig 2E).

**Table 3:** The number of transitions for 918 888 males.

	To:								
From:	Employment^a^	Sick leave	Vocational rehabilitation	Medical rehabilitation	Time limited disability pension	Disability pension	Emigrated	Dead (8)	Alive and at risk^b^
Employment^a^ (1)	0	1 657 895	45 865	48 607	3 736	20 314	28 421	10 860	729 891
Sick leave (2)	1 595 592	133 909	53 398	48 325	559	3 309	454	726	105 714
Vocational rehabilitation (3)	101 605	983	2 009	962	3 816	9 351	175	85	288
Medical rehabilitation (4)	82 070	130	0	14 381	1 320	5 164	93	81	10 566
Time limited disability benefits (5)	1 153	68	148	84	0	7 930	7	23	191
Disability benefits (6)	7 729	307	75	26	37	0	1 404	819	45 265
Emigrated (7)	26 487	317	32	51	0	46	53	13	5
Total	1 814 636	1 793 609	101 527	112 436	9 468	46 114	30 607	12 607	891 920

a Employment was defined as not being in either of the other states

b At maximum age of follow-up in 2008

For the 57 092 men with low IQ (Fig 2B), the 33 880 men with mental health problems (Fig 2D), and the 8 130 men having both low IQ and mental health problems (Fig 2F), the highest state probabilities, except for employment, at the age of 35 was for disability benefits (7.8%, 12.6%, and 18.9% respectively).

On average, men can expect to spend 22.9 years at employment in the 25-year period between the age of 20 and 45 (Table 4). For men with an average to high IQ, men without mental health problems, and men with an average to high IQ and no mental health problem, there was a higher expected period in employment (23.2, 23.1, and 23.3 respectively). For men with low IQ, men with mental health problems, and men having both low IQ and mental health problems, the mean duration of employment was less (20.6, 19.6, and 18.1 respectively). The three latter groups also had a lower duration of emigration, but longer duration of sick leave, time limited benefits, disability benefits, and lower average number of years since death (Table 4).

**Table 4:** Estimated mean number of years in the different states between 20 and 45 years of age (with 99% confidence intervals).

	Employment*	Sick leave	Time limited benefits	Disability pension	Emigrated	Dead
**All**	22.866	0.734	0.468	0.522	0.150	0.261
	(22.865–22.867)	(0.734–0.734)	(0.468–0.468)	(0.522–0.523)	(0.150–0.150)	(0.260–0.262)
**Average IQ**	23.206	0.686	0.401	0.315	0.155	0.237
**or above**	(23.205–23.206)	(0.686–0.686)	(0.401–0.401)	(0.315–0.316)	(0.155–0.155)	(0.237–0.238)
**Low IQ**	20.604	1.276	0.958	1.649	0.068	0.445
	(20.597–20.614)	(1.275–1.276)	(0.956–0.959)	(1.642–1.657)	(0.068–0.069)	(0.436–0.450)
**No mental**	23.057	0.801	0.399	0.354	0.148	0.241
**health problems**	(23.056–23.058)	(0.801–0.801)	(0.399–0.399)	(0.354–0.355)	(0.147–0.148)	(0.241–0.242)
**Mental health**	19.634	1.061	1.163	2.494	0.113	0.536
**Problems**	(19.600–19.637)	(1.059–1.061)	(1.161–1.168)	(2.487–2.521)	(0.111–0.115)	(0.526–0.554)
**Average to high IQ with**	23.266	0.749	0.351	0.260	0.151	0.224
**no mental health problems**	(23.265–23.266)	(0.749–0.749)	(0.351–0.351)	(0.259–0.261)	(0.151–0.151)	(0.224–0.225)
**Low IQ and mental**	18.085	1.186	1.422	3.732	0.068	0.507
**health problems**	(18.017–18.119)	(1.182–1.189)	(1.412–1.427)	(3.683–3.775)	(0.066–0.081)	(0.479–0.565)

* Employment was defined as not being registered in any other state

The hazard rate ratios (HRR) show the synergetic effect between IQ and mental health for the different outcomes (Table 5). Unadjusted HRR showed an increased risk for disability benefits for men with both mental health problems and low IQ compared to men with average IQ and no mental health problems both before the age of 35 years (HRR = 14.37, 95% CI [13.59-15.19]), and after 35 years of age (HRR = 16.12, 95% CI [15.27-17.01]). For the other outcomes (sick leave, time limited benefits, emigration, and death), low IQ and mental health problems had a statistical significant negative impact (Table 5).

**Table 5:** Unadjusted hazard rate ratios based on discrete time models (generalized estimation equations–GEE), showing the risk ratios with relation to mental health and IQ at military enrolment. Men with average IQ and no mental health problems are the reference group in all comparisons. There were 585 061 males with complete data for the current analyses.

		Before 35 years of age		After 35 years of age	
**Dead**		**HRR (95% CI)**	**p**	**HRR (95% CI)**	**P**
No mental problems	High IQ	0.60 (0.53–0.69)	<0.001	0.71 (0.62–0.80)	<0.001
	Average IQ	1	ref	1	Ref
	Low IQ	1.72 (1.57–1.89)	<0.001	2.11 (1.93–2.31)	<0.001
Mental problems	High IQ	1.05 (0.56–1.95)	0.89	1.27 (0.72–2.26)	0.41
	Average IQ	1.94 (1.72–2.20)	<0.001	2.21 (1.97–2.49)	<0.001
	Low IQ	2.60 (2.21–3.06)	<0.001	2.99 (2.56–3.49)	<0.001
**Emigrated**					
No mental problems	High IQ	2.49 (2.37–2.62)	<0.001	1.94 (1.80–2.09)	<0.001
	Average IQ	1	ref	1	Ref
	Low IQ	0.41 (0.36–0.47)	<0.001	0.40 (0.33–0.48)	<0.001
Mental problems	High IQ	1.93 (1.35–2.75)	<0.001	1.72 (1.12–2.66)	0.014
	Average IQ	0.83 (0.72–0.95)	0.009	0.62 (0.52–0.75)	<0.001
	Low IQ	0.40 (0.29–0.54)	<0.001	0.39 (0.27–0.56)	<0.001
**Disability benefits**					
No mental problems	High IQ	0.28 (0.24–0.31)	<0.001	0.36 (0.32–0.41)	<0.001
	Average IQ	1	ref	1	Ref
	Low IQ	4.06 (3.88–4.26)	<0.001	4.85 (4.63–5.07)	<0.001
Mental problems	High IQ	1.57 (1.09–2.26)	0.015	2.04 (1.47–2.84)	<0.001
	Average IQ	3.98 (3.73–4.24)	<0.001	4.72 (4.44–5.02)	<0.001
	Low IQ	14.37 (13.59–15.19)	<0.001	16.12 (15.27–17.01)	<0.001
**Time limited benefits**					
No mental problems	High IQ	0.32 (0.30–0.34)	<0.001	0.32 (0.30–0.35)	<0.001
	Average IQ	1	ref	1	Ref
	Low IQ	2.41 (2.35–2.48)	<0.001	2.06 (1.98–2.14)	<0.001
Mental problems	High IQ	1.49 (1.24–1.79)	<0.001	0.90 (0.67–1.19)	0.46
	Average IQ	3.03 (2.93–3.14)	<0.001	2.07 (1.96–2.17)	<0.001
	Low IQ	4.43 (4.24–4.64)	<0.001	2.70 (2.53–2.89)	<0.001
**Sick leave**					
No mental problems	High IQ	0.31 (0.30–0.32)	<0.001	0.72 (0.70–0.74)	<0.001
	Average IQ	1	ref	1	Ref
	Low IQ	1.50 (1.48–1.53)	<0.001	2.28 (2.24–2.33)	<0.001
Mental problems	High IQ	0.56 (0.48–0.64)	<0.001	1.09 (0.96–1.24)	0.20
	Average IQ	1.26 (1.23–1.29)	<0.001	1.92 (1.88–1.98)	<0.001
	Low IQ	1.48 (1.43–1.54)	<0.001	2.07 (1.99–2.15)	<0.001

Adjusting for the available physical health related parameters had a minor impact on the risk estimates for the outcomes (Table 6).

**Table 6:** Adjusted* hazard rate ratios based on discrete time models (generalized estimation equations–GEE), showing the risk ratios with relation to mental health and IQ at military enrolment. Men with average IQ and no mental health problems are the reference group in all comparisons. There were 581 941 males with complete data for the current analyses.

		Before 35 years of age		After 35 years of age	
**Dead**		**HRR (95% CI)**	**p**	**HRR (95% CI)**	**P**
No mental problems	High IQ	0.60 (0.53–0.69)	<0.001	0.71 (0.62–0.80)	<0.001
	Average IQ	1	Ref	1	Ref
	Low IQ	1.72 (1.57–1.89)	<0.001	2.12 (1.94–2.31)	<0.001
Mental problems	High IQ	1.20 (0.64–2.27)	0.57	1.47 (0.82–2.65)	0.20
	Average IQ	2.13 (1.83–2.49)	<0.001	2.44 (2.10–2.84)	<0.001
	Low IQ	2.71 (2.29–3.21)	<0.001	3.10 (2.63–3.65)	<0.001
**Emigrated**					
No mental problems	High IQ	2.48 (2.36–2.61)	<0.001	1.93 (1.79–2.08)	<0.001
	Average IQ	1	ref	1	Ref
	Low IQ	0.42 (0.36–0.48)	<0.001	0.40 (0.33–0.48)	<0.001
Mental problems	High IQ	2.40 (1.65–3.49)	<0.001	2.12 (1.33–3.38)	0.002
	Average IQ	1.00 (0.85–1.18)	0.99	0.77 (0.62–0.95)	0.016
	Low IQ	0.44 (0.32–0.60)	<0.001	0.43 (0.30–0.63)	<0.001
**Disability benefits**					
No mental problems	High IQ	0.28 (0.24–0.32)	<0.001	0.36 (0.32–0.41)	<0.001
	Average IQ	1	ref	1	Ref
	Low IQ	4.07 (3.88–4.26)	<0.001	4.85 (4.63–5.08)	<0.001
Mental problems	High IQ	1.76 (1.22–2.55)	0.003	2.29 (1.64–3.20)	<0.001
	Average IQ	4.22 (3.91–4.56)	<0.001	5.05 (4.69–5.44)	<0.001
	Low IQ	14.60 (13.76–15.49)	<0.001	16.37 (15.45–17.35)	<0.001
**Time limited benefits**					
No mental problems	High IQ	0.32 (0.30–0.34)	<0.001	0.33 (0.30–0.36)	<0.001
	Average IQ	1	Ref	1	Ref
	Low IQ	2.38 (2.32–2.44)	<0.001	2.04 (1.96–2.12)	<0.001
Mental problems	High IQ	1.37 (1.14–1.65)	0.001	0.84 (0.63–1.12)	0.23
	Average IQ	2.82 (2.70–2.95)	<0.001	1.92 (1.81–2.04)	<0.001
	Low IQ	4.16 (3.97–4.36)	<0.001	2.52 (2.35–2.70)	<0.001
**Sick leave**					
No mental problems	High IQ	0.32 (0.31–0.32)	<0.001	0.73 (0.71–0.75)	<0.001
	Average IQ	1	Ref	1	Ref
	Low IQ	1.47 (1.44–1.49)	<0.001	2.25 (2.21–2.29)	<0.001
Mental problems	High IQ	0.47 (0.40–0.54)	<0.001	0.89 (0.78–1.02)	0.092
	Average IQ	1.08 (1.05–1.11)	<0.001	1.61 (1.56–1.66)	<0.001
	Low IQ	1.34 (1.29–1.39)	<0.001	1.86 (1.78–1.93)	<0.001

* Adjusted for assessment of scores of Physical ability, Arm, Hand, Walking, Back, Skin, and BMI

## Discussion

Young men with average to high IQ without any mental health problems at military enrolment had a high probability of employment between the ages of 20 to 55. These men also had an increased chance of emigration and a low risk of disability benefits. Men with lower than average IQ or with mental health problems at the age of military enrolment, had an increased risk of long-term sick leave, time limited benefits, receiving disability benefits later in life, and early mortality. The IQ assessment was performed 1–2 years after the males had finished 9 year compulsory school. Adjustment of IQ for level of education is hence not relevant. Employment was substantially reduced for those who had both low IQ and mental health problems. Low IQ was a prominent predictor for receiving disability benefits later in life. The state occupation probabilities showed that men with mental health problems and/or low IQ had a high probability for early vocational rehabilitation before the disability benefit.

Employment is a fundamental aspect of modern society. For the individual, employment is an important part of identity, social life, personal economy, and health[33]. To what extent the society is responsible for individuals with reduced ability to work, or whether this responsibility lies with the individual, has varied over time and varies between countries[1, 2]. There is an ongoing discussion in Norway about the generous reimbursement rates and whether they contribute to long term sick leave and disability benefits[34]. The Organization for Economic Cooperation and Development (OECD) recently stated that Norway has the highest level of sick leave and costs related to lost labour among all the member countries, with common mental disorders contributing most to the recent rise in expenditures[35]. The report further concludes that the Norwegian system may encourage the exclusion of people with mental disorders through ‘welfare-traps’ of disincentives.

The precision of the evaluation of mental health at military enrolment can be questioned. However, we have shown that evaluation done for assessing ability for military duty predicts future outcomes, for men eligible to meet for the military evaluation. Whether fulfilling military service was a predictor could not be studied, since information on who actually fulfils military service was not available. Young men with too poor mental function or intellectual disabilities would not be included in the cohort as they would be too disabled to be considered for military duty. This is an important aspect to consider in the interpretation of the results[36]. Based on the regression analyses we show that IQ and mental health are approximately equally strong risk factors before and after the age of 35. However, for the time limited benefits we found that IQ and mental health were even stronger risk factors before the age of 35. This was also apparent in the figures, where the time limited benefits were bulging before the age of 35 for those with low IQ and/or mental health problems.

This study demonstrates the advantage of applying a multi-state framework for analyses of reimbursement data of multiple outcomes simultaneously. Extending the framework using time dependent factors e.g. accumulated time on sick leave or updated health information, or making causal considerations adjusting for time dependent selection is possible[37]. The effect of removing states from the system, e.g. on employment, can furthermore be evaluated[37]. The study demonstrates that IQ and mental health at young age are important risk factors for being excluded from the ordinary workforce later in life. Adolescent health problems are also markers for high school dropout[38], and high school dropouts have an increased risk for sickness and disability in young adulthood independent of own health, family and socioeconomic factors in adolescence[39].

There are limitations with the data presented in this study. The evaluation of mental health done at military enrolment cannot be regarded as diagnoses. Fadum et al. discuss the mental health evaluation done for Norwegian conscripts more in depth and also relates this to later suicide events[18]. A rapid screening of mental health is crucial before military service and there are several tools used for this screening[40]. The assessment of the physical abilities is also problematic, since all the available variables were measured with respect to the ability to perform compulsory military service. Information on those completing military service was not available, but could enhance our results further. Samuelsson et al. discusses disability pension due to mental problems and find that military service is an independent risk factor[41]. However, we still find it interesting that the evaluation done solely for the military enrolment, predicts future work drop-out.

A possible implication of the present findings could be to identify those at particularly high risk i.e. those with both low IQ and mental health problems early, and consider intervening on this high-risk group to increase integration into the labour market. Although there is no compelling evidence supporting interventions aiming at improving mental health problems in people with mild to moderate intellectual disability, there are some promising interventions that could be evaluated further in larger trials[42]. Targeted support and interventions to help cope with mental health problems and interventions like work-focused CBT and individual job support may increase work participation and prevent later adverse outcomes. Work focused CBT and individual job support have been effective in increasing or maintaining work participation for people with common mental disorders [43].

## References

[1] Jp Gonnot, Prinz C, Keilman N. Adjustments of public pension schemes in twelve industrialized countries: possible answers to population ageing. European journal of population = Revue europeenne de demographie. 1995;11(4):371–98. . 12347159 10.1007/BF01267726

[2] OECD. Mental Health and Work: Norway. Publishing O, editor: OECD Publishing; 2013.

[3] Evans-Lacko S, Knapp M. Global patterns of workplace productivity for people with depression: absenteeism and presenteeism costs across eight diverse countries. Social psychiatry and psychiatric epidemiology. 2016;51(11):1525–37. doi: 10.1007/s00127-016-1278-4 . 27667656 10.1007/s00127-016-1278-4 PMC5101346

[4] Henderson M, Harvey SB, Overland S, Mykletun A, Hotopf M. Work and common psychiatric disorders. Journal of the Royal Society of Medicine. 2011;104(5):198–207. doi: 10.1258/jrsm.2011.100231 . 21558098 10.1258/jrsm.2011.100231 PMC3089873

[5] Henderson M, Richards M, Stansfeld S, Hotopf M. The association between childhood cognitive ability and adult long-term sickness absence in three British birth cohorts: a cohort study. BMJ open. 2012;2(2):e000777 doi: 10.1136/bmjopen-2011-000777 . 22466159 10.1136/bmjopen-2011-000777 PMC3323804

[6] Pape K, Bjorngaard JH, Holmen TL, Krokstad S. The welfare burden of adolescent anxiety and depression: a prospective study of 7500 young Norwegians and their families: the HUNT study. BMJ open. 2012;2(6):e001942 doi: 10.1136/bmjopen-2012-001942 . 23144262 10.1136/bmjopen-2012-001942 PMC3533058

[7] Bratberg E, Gjesdal S, Maeland JG. Sickness absence with psychiatric diagnoses: Individual and contextual predictors of permanent disability. Health & Place. 2009;15(1):308–14. doi: 10.1016/j.healthplace.2008.06.004 18692427 10.1016/j.healthplace.2008.06.004

[8] Gravseth HM, Bjerkedal T, Irgens LM, Aalen OO, Selmer R, Kristensen P. Life course determinants for early disability pension: a follow-up of Norwegian men and women born 1967–1976. Eur J Epidemiol. 2007;22(8):533–43. doi: 10.1007/s10654-007-9139-9 . 17530421 10.1007/s10654-007-9139-9

[9] Henderson M, Stansfeld S, Hotopf M. Self-rated health and later receipt of work-related benefits: evidence from the 1970 British Cohort Study. Psychological medicine. 2013;43(8):1755–62. doi: 10.1017/S0033291712002528 . 23137468 10.1017/S0033291712002528

[10] Stover M, Pape K, Johnsen R, Fleten N, Sund ER, Claussen B, et al Unemployment and disability pension—an 18-year follow-up study of a 40-year-old population in a Norwegian county. BMC public health. 2012;12:148 doi: 10.1186/1471-2458-12-148 . 22369630 10.1186/1471-2458-12-148 PMC3305666

[11] Gjesdal S, Haug K, Ringdal P, Maeland JG, Hagberg J, R²raas T, et al Sickness absence with musculoskeletal or mental diagnoses, transition into disability pension and all-cause mortality: a 9-year prospective cohort study. Scand J Public Health. 2009;37(4):387–94. doi: 10.1177/1403494809103994 . 19324926 10.1177/1403494809103994

[12] Gjesdal S, Ringdal PR, Haug K, Maeland JG, Vollset SE, Alexanderson K. Mortality after long-term sickness absence: prospective cohort study. Eur J Public Health. 2008;18(5):517–21. doi: 10.1093/eurpub/ckn010 . 18332039 10.1093/eurpub/ckn010

[13] van der Noordt M, Ijzelenberg H, Droomers M, Proper KI. Health effects of employment: a systematic review of prospective studies. Occupational and environmental medicine. 2014;71(10):730–6. doi: 10.1136/oemed-2013-101891 . 24556535 10.1136/oemed-2013-101891

[14] Rueda S, Chambers L, Wilson M, Mustard C, Rourke SB, Bayoumi A, et al Association of returning to work with better health in working-aged adults: a systematic review. American journal of public health. 2012;102(3):541–56. doi: 10.2105/AJPH.2011.300401 . 22390520 10.2105/AJPH.2011.300401 PMC3487667

[15] Batty GD, Deary IJ, Gottfredson LS. Premorbid (early life) IQ and later mortality risk: systematic review. Annals of epidemiology. 2007;17(4):278–88. doi: 10.1016/j.annepidem.2006.07.010 . 17174570 10.1016/j.annepidem.2006.07.010

[16] Sorberg A, Lundin A, Allebeck P, Melin B, Falkstedt D, Hemmingsson T. Cognitive ability in late adolescence and disability pension in middle age: follow-up of a national cohort of Swedish males. PloS one. 2013;8(10):e78268 doi: 10.1371/journal.pone.0078268 . 24147128 10.1371/journal.pone.0078268 PMC3797835

[17] "Instruction for military health-service and medical evaluation" (Bestemmelse for militær helsetjeneste og legebedømmelse—FSAN P6). 2013.

[18] Fadum EA, Fonnebo V, Borud EK. Presence of minor and major mental health impairment in adolescence and death from suicide and unintentional injuries/accidents in men: a national longitudinal cohort study. Journal of epidemiology and community health. 2017;71(1):19–24. doi: 10.1136/jech-2016-207656 . 27417429 10.1136/jech-2016-207656

[19] Sundet JM, Barlaug DG, Torjussen TM. The end of the Flynn effect? A study of secular trends in mean intelligence test scores of Norwegian conscripts during half a century. Intelligence. 2004;32(4):349–62. doi: 10.1016/j.intell.2004.06.004

[20] Brinch CN, Galloway TA. Schooling in adolescence raises IQ scores. Proceedings of the National Academy of Sciences of the United States of America. 2012;109(2):425–30. doi: 10.1073/pnas.1106077109 22203952 10.1073/pnas.1106077109 PMC3258640

[21] Lie SA, Eriksen HR, Ursin H, Hagen EM. A multi-state model for sick-leave data applied to a randomized control trial study of low back pain. Scand J Public Health. 2008;36(3):279–83. doi: 10.1177/1403494807086979 . 18519297 10.1177/1403494807086979

[22] Pedersen J, Bjorner JB, Burr H, Christensen KB. Transitions between sickness absence, work, unemployment, and disability in Denmark 2004–2008. Scand J Work Environ Health. 2012;38(6):516–26. doi: 10.5271/sjweh.3293 . 22441355 10.5271/sjweh.3293

[23] Oyeflaten I, Lie SA, Ihlebak CM, Eriksen HR. Multiple transitions in sick leave, disability benefits, and return to work.—A 4-year follow-up of patients participating in a work-related rehabilitation program. BMC public health. 2012;12:748 doi: 10.1186/1471-2458-12-748 . 22954254 10.1186/1471-2458-12-748 PMC3490737

[24] Andersen PK, Keiding N. Multi-state models for event history analysis. Stat Methods Med Res. 2002;11(2):91–115. doi: 10.1191/0962280202SM276ra . 12040698 10.1191/0962280202SM276ra

[25] Commenges D. Inference for multi-state models from interval-censored data. Stat Methods Med Res. 2002;11(2):167–82. doi: 10.1191/0962280202sm279ra . 12040695 10.1191/0962280202sm279ra

[26] Aalen OO, Johansen S. Empirical Transition Matrix for Nonhomogeneous Markov-Chains Based on Censored Observations. Scand J Stat. 1978;5(3):141–50.

[27] Datta S, Satten GA. Estimation of integrated transition hazards and stage occupation probabilities for non-Markov systems under dependent censoring. Biometrics. 2002;58(4):792–802. . 12495133 10.1111/j.0006-341x.2002.00792.x

[28] Gunnes N, Borgan O, Aalen OO. Estimating stage occupation probabilities in non-Markov models. Lifetime Data Anal. 2007;13(2):211–40. doi: 10.1007/s10985-007-9034-4 . 17334924 10.1007/s10985-007-9034-4

[29] Andersen PK, Pohar Perme M. Inference for outcome probabilities in multi-state models. Lifetime data analysis. 2008;14(4):405–31. doi: 10.1007/s10985-008-9097-x . 18791824 10.1007/s10985-008-9097-x PMC2735091

[30] Steele F. Multilevel discrete-time event history models with applications to the analysis of recurrent employment transitions. Aust NZJStat. 2011;53(1):1–26.

[31] Andersen PK, Klein JP, Rosthoj S. Generalised linear models for correlated pseudo-observations, with applications to multi-state models. Biometrika. 2003;90(1):15–27. doi: 10.1093/biomet/90.1.15

[32] Andersen PK, Perme MP. Pseudo-observations in survival analysis. Stat Methods Med Res. 2010;19(1):71–99. doi: 10.1177/0962280209105020 . 19654170 10.1177/0962280209105020

[33] Gravseth HM, Bjerkedal T, Irgens LM, Aalen OO, Selmer R, Kristensen P. Influence of physical, mental and intellectual development on disability in young Norwegian men. European journal of public health. 2008;18(6):650–5. doi: 10.1093/eurpub/ckn055 . 18593730 10.1093/eurpub/ckn055

[34] Bratsberg B, Fevang E, Roed K. Job loss and disability insurance. Labour Economics. 2013;24:137–50. doi: 10.1016/j.labeco.2013.08.004

[35] OECD. Reconsidering Norwegian sickness absence policies, in Mental Health and Work: Norway. Publishing O, editor: OECD Publishing; 2013.

[36] Moster D, Lie RT, Markestad T. Long-term medical and social consequences of preterm birth. The New England journal of medicine. 2008;359(3):262–73. doi: 10.1056/NEJMoa0706475 . 18635431 10.1056/NEJMoa0706475

[37] Gran JM, Lie SA, Oyeflaten I, Borgan O, Aalen OO. Causal inference in multi-state models-sickness absence and work for 1145 participants after work rehabilitation. BMC Public Health. 2015;15(1):1082 doi: 10.1186/s12889-015-2408-8 ; PubMed Central PMCID: PMC4619267. 26498223 10.1186/s12889-015-2408-8 PMC4619267

[38] De Ridder KAA, Pape K, Johnsen R, Holmen TL, Westin S, Bjorngaard JH. Adolescent health and high school dropout: a prospective cohort study of 9000 Norwegian adolescents (the young-HUNT). PloS one. 2013;8(9):e74954 doi: 10.1371/journal.pone.0074954 . 24086408 10.1371/journal.pone.0074954 PMC3781164

[39] De Ridder KAA, Pape K, Cuypers K, Johnsen R, Holmen TL, Westin S, et al High school dropout and long-term sickness and disability in young adulthood: a prospective propensity score stratified cohort study (the Young-HUNT study). BMC public health. 2013;13:941 doi: 10.1186/1471-2458-13-941 . 24103558 10.1186/1471-2458-13-941 PMC4124891

[40] Cardona RA, Ritchie EC. U.S. military enlisted accession mental health screening: history and current practice. Mil Med. 2007;172(1):31–5. . 17274262 10.7205/milmed.172.1.31

[41] Samuelsson A, Ropponen A, Alexanderson K, Svedberg P. Psychosocial working conditions, occupational groups, and risk of disability pension due to mental diagnoses: a cohort study of 43,000 Swedish twins. Scand J Work Environ Health. 2013;39(4):351–60. doi: 10.5271/sjweh.3338 . 23248027 10.5271/sjweh.3338

[42] Koslowski N, Klein K, Arnold K, Kosters M, Schutzwohl M, Salize HJ, et al Effectiveness of interventions for adults with mild to moderate intellectual disabilities and mental health problems: systematic review and meta-analysis. Br J Psychiatry. 2016 doi: 10.1192/bjp.bp.114.162313 . 27198481 10.1192/bjp.bp.114.162313

[43] Reme SE, Grasdal AL, Lovvik C, Lie SA, Overland S. Work-focused cognitive-behavioural therapy and individual job support to increase work participation in common mental disorders: a randomised controlled multicentre trial. Occup Environ Med. 2015;72(10):745–52. doi: 10.1136/oemed-2014-102700 ; PubMed Central PMCID: PMCPMC4602235. 26251065 10.1136/oemed-2014-102700 PMC4602235

